# Temporal trend of microenvironmental time-activity patterns of the Seoul population from 2004 to 2022 and its potential impact on exposure assessment

**DOI:** 10.1038/s41370-024-00662-1

**Published:** 2024-03-28

**Authors:** Donghyun Kim, Sooyoung Guak, Kiyoung Lee

**Affiliations:** 1 Institute of Health and Environment, Seoul National University, 1 Gwanak-ro, Gwanak-gu, Seoul 08826 Korea; 2 Department of Environmental Health Sciences, Graduate School of Public Health, Seoul National University, 1 Gwanak-ro, Gwanak-gu, Seoul 08826 Korea

**Keywords:** Time-activity pattern, microenvironment, temporal trend, sociodemographic variation, exposure modeling, exposure assessment

## Abstract

**Background:**

Time-activity pattern (TAP) is an important parameter for determining personal exposure to environmental pollutants. Changes in TAPs could have significant implications for the alterations in outcomes of exposure assessments.

**Objective:**

This study aimed to evaluate the Seoul population’s long-term change in TAPs, along with variations by sociodemographic group.

**Methods:**

In 2004, 2009, 2014, and 2019, the Time Use Survey of Statistics Korea collected the TAP information of 4036, 2610, 3337, and 2793 Seoul residents, respectively. In 2022, the TAP information of 4401 Seoul residents was collected for Korean Air Pollutant Exposure (KAPEX) research. The microenvironmental TAP changes in the Seoul population from 2004 to 2022 were assessed based on age, gender, work status, and day type.

**Results:**

From 2004 to 2022, Seoul people increasingly spent more time in indoor residences (from 14.8 ± 5.1 h to 15.8 ± 4.5 h) and less time in other indoors (from 7.2 ± 4.5 h to 5.9 ± 4.2 h). Their transit time constantly decreased from 2004 (1.4 ± 1.8 h) to 2022 (1.2 ± 1.3 h), whereas the outdoor time fluctuated throughout the years. From 2004 to 2022, the time of the day spent by Seoul people in residential indoor shifted to later in the morning (2004: 8:30 am; 2022: 9:00 am) and earlier in the evening (2004: 9:30 pm; 2022: 7:00 pm); however, the opposite was true for other indoors (2004: from 8:30 am to 9:30 pm; 2022: from 9:00 am to 7:00 pm) and transits (2004: 7:30–9:30 am and 3:00–8:00 pm; 2022: 7:30–9:00 pm and 5:00–9:00). The time of the day spent in outdoors increased from 2004 to 2019, with a distinct peak observed in 2022 (12:00 pm–2:00 pm). The microenvironmental time trends of adolescents and late-adulthoods differed from those of the other age groups, while those of males differed from females. Also, the microenvironmental time trends of the employed differed from those of the unemployed, and those during weekdays differed from those during weekends.

**Impact statement:**

Microenvironmental TAP should be essentially considered to estimate the actual exposure to pollutants. This study demonstrates the Seoul population’s long-term changes in TAP throughout the 18 years as the significant parameter in exposure assessment. Notably, the microenvironmental TAPs of Seoul people shifted, with variations across different sociodemographic groups. Previous studies in Korea did not consider the TAP shifts in exposure assessment; this study highlights the importance of aligning TAP data with concurrent environmental pollutant data and emphasizes the need for refined data collection in future exposure assessments.

## Introduction

Time-activity pattern (TAP) represents the allocation of an individual’s time and activities at different locations. As people move to different microenvironments with different pollutant concentrations, TAP information is crucial for estimating total human exposure [1]. Exposure to environmental pollutants is equal to the concentration of pollutants multiplied by the duration in the locations. Therefore, exposure estimation process involves: (i) collection of TAP data comprised of an individual’s duration information in various microenvironments including residential indoor, other indoors, transit, and outdoor, (ii) integration of the TAP data into exposure models, combining the microenvironmental time spent with microenvironment-specific pollutant concentration for calculation of individual’s exposures in each setting, and (iii) aggregation of the calculations across all microenvironments for providing a comprehensive estimation of the individual’s total exposure over the given period [2–4]. In Korea, the TAP information of 3984 people in Seoul was matched with the microenvironmental concentration of fine particulate matter (i.e., particles <2.5 μm aerodynamic diameter; PM_2.5_) for the estimation of population exposures to PM_2.5_ [2]. Similarly, studies in the U.S. incorporated the TAP information of more than 22,000 people with microenvironmental PM_2.5_ concentrations into stochastic models for estimating population exposures [3, 4].

TAP information has been collected on a national scale in several countries. In the U.S., the National Human Activity Pattern Survey (NHAPS) was a single study conducted from 1992 to 1994, that comprehensively investigated the TAPs of 9386 participants in 48 contiguous states: in addition to the 25 min telephone survey, a 24 h time-activity survey with an exposure-specific activity questionnaire diary (e.g., time-location, sports-related activities, painting, and auto repair) was conducted [5]. The Consolidated Human Activity Database (CHAD) pooled time-activity data from 23 independent exposure and time-use studies in the U.S., incorporating 179,250 participants’ diary-days information [6, 7]. In Canada, the Canadian Human Activity Pattern Survey (CHAPS) II collected time-location data from 5011 participants using telephone interview and questionnaire survey [8]. To reflect the effect of societal trends, TAPs were collected by age, season, and gender. In Europe, the Exposure to Air Pollution in a Southern European City (EXPOLIS) collected TAP data of 1447 participants from Helsinki, Athens, Basel, Grenoble, Milan, Prague, and Oxford over four years [9]. The subjects recorded a 48 h time-location diary every 15 min, which consisted of location questionnaires for residential indoors, working indoors, other indoors, and indoors exposed to environmental tobacco smoke.

The Time Use Survey of Statistics Korea (KOSTAT) collected TAP information for Koreans every five years since 1999 [10]. Each survey collected a 24 h activity pattern diary of >21,000 subjects selected from 17 provinces and metropolitan cities in Korea. However, the TAP survey was aimed to investigate the social and economic status of Koreans. To provide preliminary information relevant to exposure assessment, Yang et al. (2011) reclassified the TAP data of 31,634 participants collected in 2004 into time-location data and determined the sociodemographic factors affecting the TAPs [11]. In another study, the TAP data of 31,634 and 20,263 participants obtained in 2004 and 2009, respectively, were analyzed to classify the population according to the activity patterns necessary for exposure assessment [12]. In a follow-up study, researchers examined the TAP data of 26,988 participants collected in 2014 to identify the effect of season on time-activity [13].

TAP could be affected by various factors. Several studies have demonstrated that demographic characteristics—including age, gender, work status, and day type (e.g., weekdays and weekends)—were the most influential factors affecting individuals’ time-location patterns. Compared with CHAPS I from the early 1990s, in CHAPS II from 2010 to 2011, the average residential indoor time spent by adolescents increased by 24 min, whereas that of adults increased by 1 h 13 min [8]. In addition, gender was another significant predictor of TAP in that study: males spent more time outdoors than females, whereas the opposite was true for residential indoors. In a study in Korea, the microenvironmental time spent by workers significantly differed from that of other population groups, especially those that were jobless [14]. Similarly, day type significantly affected TAP in a study in the U.S.[6].

TAP may have changed over time. Changes in TAPs may have important implications for changes in exposure to environmental pollutants; however, no studies in Korea have comprehensively assessed the long-term change in microenvironmental TAPs. Fragmentary studies in Korea have analyzed TAPs collected in 2004, 2009, and 2014, but these studies have not reflected sociodemographic changes over time [11–13]. A study in Canada compared the TAP data retrieved from CHAPS I and CHAPS II [8]. However, the study had a 15 year gap between the surveys, providing a fragmented comparison of the TAP data. In particular, the advent of the Coronavirus disease-2019 (COVID-19) have significantly affected people’s lifestyles. With lockdowns and work-from-home policies, the COVID-19 pandemic resulted in longer hours spent in residential indoors [15]. In a U.S. study, sedentary time increased by approximately 41% compared to pre-COVID-19 levels [16].

This study aimed to evaluate the long-term trend of Seoul population’s microenvironmental TAPs from 2004 to 2022. The time spent by the Seoul population at different microenvironments in 2004, 2009, 2014, 2019, and 2022 was analyzed to identify the change in TAPs by demographic and sociodemographic factors including age, gender, work status, and day type.

## Materials and methods

### Time use survey of Statistics Korea (KOSTAT) data

The Time Use Survey of Statistics Korea (KOSTAT) collected the TAP information of 32,000, 21,000, 27,000, and 29,000 subjects from 17 provinces and metropolitan cities in Korea in 2004, 2009, 2014, and 2019, respectively [10]. The study population were selected from 800 to 850 areas of Korea, and stratified classification was used to choose fifteen households in each area. Households were selected via systematically extracting points at regular intervals within each survey area, with the regional and housing type characteristics in consideration. For each survey area, stratification criteria were established based on gender and age groups, and the sample was allocated to reflect the actual composition ratio. In each household, residents above 10 years old who agreed to participate were recruited. The TAP information of 4036, 2610, 3337, and 2793 subjects collected from Seoul in 2004, 2009, 2014, and 2019, respectively, was selected for this study.

The survey questionnaire consisted of nine main categories of sociodemographic questions: (i) personal care management; (ii) occupation; (iii) education level; (iv) household chore activity; (v) family caregiving; (vi) volunteer activity or internship; (vii) social activity; (viii) leisure activity; and (ix) transportation. The nine main categories were subdivided into 50 subcategories, which were further categorized into >120 items (Table S1). The time-location was classified by the following microenvironments: (1) time spent at residential indoor, other indoors, using transportation, and outdoors in 2004 and 2009; (2) time spent at residential indoor, workplace or school, restaurant or bar, other indoors, using transportation (bus, subway, train, taxi, private vehicle, and etc.), and outdoors in 2014 and 2019.

The data was collected using a face-to-face survey method via trained researchers, which involved two steps: (i) interview surveys and (ii) time-diary methods [10, 17]. Participants aged 10 and above recorded their activities and time-location information in a diary every 10 min for 48 h period (Fig. S1). Before initiating the surveys, respondents were instructed by the researchers to ensure that no time-diary entries were to be omitted. Researchers immediately reviewed the collected survey forms on-site for any inconsistencies, missing responses, and errors. Any discrepancies or omissions were rectified via follow-up visits or phone calls. Any part of the survey form requiring correction was marked for review and processed according to the protocol. After completing the reviews, the researchers assigned activity and location classification codes on the data provided by the respondents.

A total of 25,552 person-days diary data of the Seoul population were collected during weekdays and weekends in 2004, 2009, 2014, and 2019, respectively. In 2004, 8072 person-days data were collected during September on weekdays (4848 person-days) and weekends (3224 person-days). In 2009, 3218 and 2002 person-days data were collected during March and September, respectively, on weekdays (3113 person-days) and weekends (2107 person-days). In 2014, 1628, 3204, and 1842 person-days data were collected during July, September, and December, respectively, on weekdays (3984 person-days) and weekends (2690 person-days). In 2019, 1572, 2252, and 1762 person-days data were collected during July, September, and December, respectively, on weekdays (3388 person-days) and weekends (2198 person-days). For comparison, person-day data collected in September were extracted for this study.

### Korean air pollutant exposure model (KAPEX) research data

The TAP information of 4401 subjects were collected from Seoul during 2022 for the Korean Air Pollutant Exposure model (KAPEX) project. Survey districts were selected using the enumeration data from KOSTAT. Households were selected via systematically extracting points at regular intervals within each survey area, with the regional and housing type characteristics in consideration. For each survey area, stratification criteria were established based on gender and age groups and the sample was allocated to reflect the actual composition ratio. In each household, residents above 10 years old who agreed to participate were recruited.

The survey questionnaire consisted of demographic characteristics (gender, age, etc.), personal characteristics (height, weight, medical issues, etc.), and socioeconomic characteristics (occupation, housing type, income, educational level, etc.). Time-location was classified by the following microenvironments: (i) residential indoor; (ii) workplace or school; (iii) kindergarten; (iv) restaurant; (v) other eating places (café and bar); (vi) study places (private educational institute, library, study café, etc.); (vii) shopping places (shopping mall, market, department store); (viii) leisure places (PC room, billiard club, screen golf club, etc.); (ix) other places; (x) outdoor; and (xi) transportation (bus, subway, train, taxi, private vehicle, etc.).

Following the steps established by KOSTAT, data was collected via trained researchers using a face-to-face survey method that involved (i) interview surveys and (ii) time-diary methods. In addition, mobile and online platforms were utilized to assist in recording the 24 h activities. Participants aged 10 and above recorded their activities and time-location information in a diary every 10 min for 48 h period. In accordance to the protocol established in KOSTAT, researchers instructed participants on filling out time-diaries, and promptly addressed any errors or inconsistencies in the completed surveys through immediate checks and follow-up actions.

Diary data for 8766 person-days of the Seoul population were collected: 2194, 2202, 2172, and 2198 person-days were collected during March, July, September, and December in 2022, respectively, on weekdays (4403 person-days) and weekends (4363 person-days). For comparison, person-day data collected in September were extracted for this study.

### Data extraction and curation

The microenvironment information collected in 2004, 2009, 2014, 2019, and 2022 is summarized and reclassified in Table 1. The microenvironmental classification of the KOSTAT survey in 2004 and 2009 included information of residential indoors, other indoors, transportations, and outdoors; since further subdivision was unlikely, microenvironmental data from 2014, 2019, and 2022 were reclassified into four microenvironments. The microenvironments investigated in 2014 and 2019 were reclassified as follows: (i) residential indoor included occupation in residence; (ii) workplace or school, restaurant or bar, and other indoors were classified as other indoors; (iii) using bus, subway, train, taxi, private vehicle, etc. was classified as using transportation; and (iv) outdoors included all outdoor places. Microenvironments investigated in 2022 were reclassified as follows: (i) residential indoor included occupation in residence; (ii) workplace or school, kindergarten, restaurant, other eating places (café and bar), study places (private educational institute, library, study café, and etc.), shopping places (shopping mall, market, department store), leisure places (PC room, billiard club, screen golf club, and etc.), and other places were classified as other indoors; (iii) using bus, subway, train, taxi, private vehicle, and etc. was classified as using transportation; and (iv) outdoors included all outdoor places.

**Table 1:** Microenvironmental information collected from 2004 to 2022.

Investigation year	Microenvironments investigated in each year	
	Main categories	Subcategories^*^
2004	Residential indoor	–
	Other indoor	–
	Transportation	–
	Outdoor	–
2009	Residential indoor	–
	Other indoor	–
	Transportation	–
	Outdoor	–
2014	Residential indoor	–
	Other indoor	Workplace, school, restaurant, bar, and etc.^a^
	Transportation	Bus, subway, train, taxi, private vehicle, and etc.^b^
	Outdoor	–
2019	Residential indoor	–
	Other indoor	Workplace, school, restaurant, bar, and etc.^a^
	Transportation	Bus, subway, train, taxi, private vehicle, and etc.^b^
	Outdoor	–
2022	Residential indoor	–
	Other indoor	Workplace, school, kindergarten, restaurant, cafe, bar, private educational institute, library, study cafe, shopping mall, market, department store, PC room, billiard club, screen golf club and etc.^**c**^
	Transportation	Bus, subway, train, taxi, private vehicle, and etc.^b^
	Outdoor	–

*Only the microenvironments that were subcategorized from the main categories are listed. Microenvironments that were not further subdivided were investigated by main categories.

^a^‘etc.’ under ‘Other indoor’ category in 2014 and 2019 refers to additional other indoor locations not included in the four sub-classified microenvironments.

^b^‘etc.’ under ‘Transportation’ category in 2014, 2019, and 2022 refers to additional types of transportation not included in the five sub-classified transit types.

^c^‘etc.’ under ‘Other indoor’ category in 2022 refers to additional other indoor locations not included in the fourteen sub-classified microenvironments.

### Statistical analysis

Across the years, data collection seasons varied (September in 2004; March and September in 2009; March, September, and December in 2014 and 2019; March, July, September, and December in 2022), with only September being consistently represented. Despite the notable seasonal variations in TAP characteristics, due to limitations in cross-seasonal comparisons and to ensure comparability, September data collected in different years were selected for comparison in this study. To check the normality of distribution, the Kolmogorov-Smirnov test was conducted on the data collected in 2004, 2009, 2014, 2019, and 2022. As all tests showed statistically normal distributions (*p* < 0.001), arithmetic mean (AM) and standard deviation (SD) were used. Analysis of variance (ANOVA) was employed to determine the difference in the amount of time spent in each microenvironment in different years. For the post-hoc test, Scheffé’s method was used to evaluate the statistical difference between the microenvironmental time spent in each year. All statistical analyses were conducted using Rex-software version 3.3.1.1 (Rexsoft, Co. Ltd., Seoul, KR). A *p*-value of <0.05 was considered statistically significant.

## Results

### Microenvironmental time spent by Seoul population from 2004 to2022

The time spent by the Seoul population in the four microenvironments from 2004 to 2022 is presented in Table 2. The time spent by the Seoul population in residential indoors significantly increased from 14.8 ± 5.1 h in 2004 to 15.8 ± 4.5 h in 2022 (*p* < 0.01), except from 15.3 ± 4.8 h in 2009 to 15.1 ± 4.9 h in 2014 (*p* = 0.079), which slightly decreased. In contrast, the time spent by the Seoul population in other indoors significantly decreased from 7.2 ± 4.5 h in 2004 to 5.9 ± 4.2 h in 2022 (*p* < 0.001), except from 6.8 ± 4.2 h in 2009 to 7.1 ± 4.3 h in 2014 (*p* = 0.741), which slightly increased. The time spent in transit by the Seoul population constantly decreased from 1.4 ± 1.8 h in 2004 to 1.2 ± 1.3 h in 2022 (*p* < 0.05). The time spent outdoors by the Seoul population significantly increased from 0.6 ± 0.6 h in 2004 to 1.1 ± 1.8 h in 2022 (*p* < 0.001), except from 0.7 ± 0.7 h in 2009 to 0.5 ± 0.6 h in 2014 (*p* = 0.142), which slightly decreased.

**Table 2:** Descriptive statistics of the Seoul population’s time-location from 2004 to 2022.

Demographic characteristic	Microenvironment	Year^a^					*p* value^b^
		2004 (*n* = 8072)	2009 (*n* = 2002)	2014 (*n* = 3204)	2019 (*n* = 2252)	2022 (*n* = 2172)	
Seoul population	Residential Indoor	14.8 ± 5.1	15.3 ± 4.8	15.1 ± 4.9	15.6 ± 4.8	15.8 ± 4.5	<0.001
	Other Indoors	7.2 ± 4.5	6.8 ± 4.2	7.1 ± 4.3	6.0 ± 4.4	5.9 ± 4.2	<0.001
	Transportation	1.4 ± 1.8	1.3 ± 1.6	1.3 ± 1.3	1.2 ± 1.3	1.2 ± 1.3	<0.001
	Outdoor	0.6 ± 0.6	0.7 ± 0.7	0.5 ± 0.6	1.1 ± 1.4	1.1 ± 1.8	<0.001

^a^The data collected in September are compared. The microenvironmental time spent (h) is given as arithmetic mean (AM) ± standard deviation (SD).

^b^Statistical significance of the differences in time spent within each microenvironment from 2004 to 2022, as determined by ANOVA test.

### Time-location profiles of Seoul population from 2004 to 2022

**Fig. 1:**
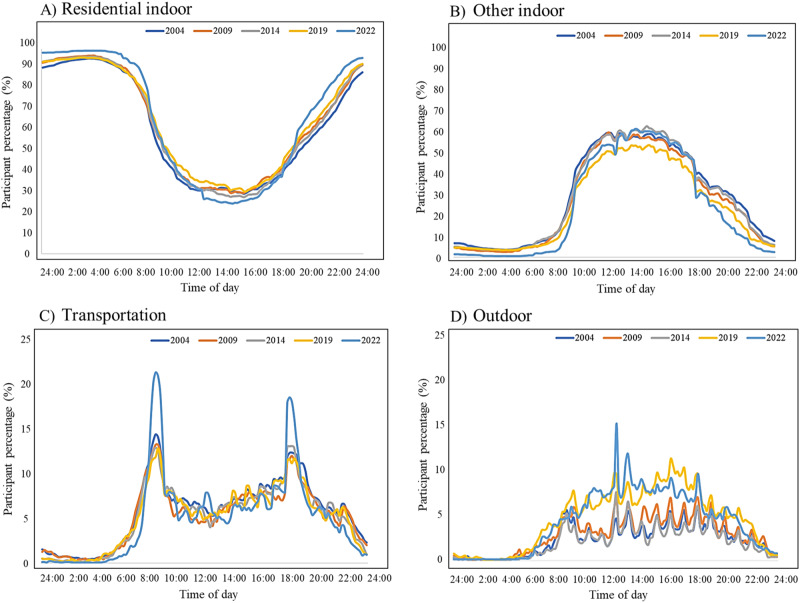
The time-location profiles of the Seoul population (%) in residential indoors, other indoors, transits, and outdoors from 2004 to 2022. **A** Is the hourly profiles in residential indoors, **B** is the hourly profiles in other indoors, (**C**) is the hourly profiles in transits, and (**D**) is the hourly profiles in outdoors.

The time-location profiles of the Seoul population in the four microenvironments from 2004 to 2022 are presented in Fig. 1. The time of the day spent in residential indoors by >75% of participants gradually increased from 2004 (10:30 pm to 7:30 am) to 2022 (8:30 pm to 8:00 am). In contrast, the time of the day spent by >25% of participants in other indoors gradually decreased from 2004 (8:30 am to 9:30 pm) to 2022 (9:00 am to 7:00 pm). The time of the day spent in transits by >8% of participants gradually decreased from 2004 (7:30 am to 9:30 am and 3:00 pm to 8:00 pm) to 2022 (7:30 am to 9:00 am and 5:00 pm to 7:30 pm), but the proportion of participants increased in 2022. The time of the day spent outdoors by the participants gradually increased from 2004 to 2019, with a peak observed from 12:00 pm to 2:00 pm in 2022.

### TAP trends by demographic groups from 2004 to 2022

The time spent by different age groups and genders in the four microenvironments from 2004 to 2022 is presented in Table 3. For most age groups, the TAP trends in the four microenvironments were similar throughout the period. However, the residential indoor time spent by adolescents decreased from 14.7 ± 4.1 h in 2004 to 14.5 ± 4.2 h in 2009 (*p* = 0.751), whereas that of late-adulthoods remained relatively unchanged from 18.0 ± 4.4 h in 2014 to 18.0 ± 4.2 h in 2022 (*p* > 0.1). The general trend of the other indoor time spent by adolescents increased from 7.9 ± 3.7 h in 2004 to 8.0 ± 3.8 h in 2009 (*p* = 0.430) and decreased from 8.0 ± 3.8 h in 2009 to 5.2 ± 4.1 h in 2022 (*p* > 0.1). The other indoor time spent by late-adulthoods was significantly shorter in 2009 (4.3 ± 3.2 h; *p* < 0.05) and 2019 (3.6 ± 3.8 h; *p* < 0.001) than in 2004, 2014, and 2022 (4.8 ± 4.4 h, 4.6 ± 3.8 h, and 3.9 ± 4.0 h, respectively). The outdoor time spent by late-adulthoods increased the most from 0.6 ± 0.6 h in 2014 to 1.6 ± 1.5 h in 2019 (*p* < 0.001) and was up to five times higher than those of other age groups. The residential indoor time spent by males, unlike that spent by females, increased constantly from 14.0 ± 4.7 h in 2009 to 15.2 ± 4.6 h in 2022 (*p* < 0.05), whereas the transit time decreased from 1.8 ± 2.1 h in 2004 to 1.4 ± 1.3 h in 2019 (*p* < 0.05).

**Table 3:** Descriptive statistics of the microenvironmental time spent by different demographic groups from 2004 to 2022.

Demographic characteristic		Microenvironment	Year^a^					*p*-value^b^
			2004	2009	2014	2019	2022	
**Age**	Adolescent (<19)	Number of participants	1208	176	392	186	312	
		Residential Indoor	14.7 ± 4.1	14.5 ± 4.2	15.0 ± 4.4	15.9 ± 4.5	17.3 ± 4.4	0.001
		Other Indoors	7.9 ± 3.7	8.0 ± 3.8	7.5 ± 4.0	6.4 ± 4.1	5.2 ± 4.1	<0.001
		Transportation	0.7 ± 1.0	0.7 ± 0.9	0.8 ± 1.0	0.8 ± 1.0	0.7 ± 0.9	0.009
		Outdoor	0.8 ± 0.6	0.9 ± 0.6	0.7 ± 0.6	0.9 ± 1.0	0.8 ± 1.2	<0.001
	Early adulthood (19-29)	Number of participants	1638	244	450	352	337	
		Residential Indoor	13.8 ± 5.0	14.2 ± 4.8	14.0 ± 4.7	14.5 ± 5.2	15.1 ± 4.7	<0.001
		Other Indoors	8.0 ± 4.4	7.7 ± 4.2	7.8 ± 4.1	7.4 ± 4.6	6.7 ± 4.2	<0.001
		Transportation	1.6 ± 1.5	1.5 ± 1.2	1.5 ± 1.4	1.4 ± 1.2	1.3 ± 1.1	0.006
		Outdoor	0.6 ± 0.6	0.6 ± 0.5	0.6 ± 0.6	0.8 ± 1.1	0.8 ± 1.4	<0.001
	Mid adulthood (30-49)	Number of participants	3318	870	1146	764	660	
		Residential Indoor	14.5 ± 5.3	14.9 ± 4.9	14.7 ± 5.2	15.5 ± 5.0	16.1 ± 4.8	<0.001
		Other Indoors	7.4 ± 4.7	7.1 ± 4.3	7.3 ± 4.6	6.3 ± 4.5	5.8 ± 4.4	<0.001
		Transportation	1.6 ± 1.8	1.4 ± 1.6	1.5 ± 1.4	1.5 ± 1.3	1.3 ± 1.3	0.01
		Outdoor	0.5 ± 0.5	0.6 ± 0.6	0.5 ± 0.5	0.8 ± 1.1	0.8 ± 1.6	<0.001
	Mid-late adulthood (50-64)	Number of participants	1314	458	734	582	543	
		Residential Indoor	15.3 ± 5.0	15.6 ± 4.6	15.3 ± 4.9	15.3 ± 5.0	16.2 ± 4.5	0.006
		Other Indoors	6.5 ± 4.3	6.3 ± 3.9	6.9 ± 4.3	6.3 ± 4.6	5.6 ± 4.2	<0.001
		Transportation	1.6 ± 2.2	1.4 ± 1.9	1.2 ± 1.3	1.4 ± 1.4	1.2 ± 1.4	0.002
		Outdoor	0.6 ± 0.6	0.7 ± 0.6	0.5 ± 0.5	1.1 ± 1.4	1.0 ± 1.5	<0.001
	Late adulthood (>65)	Number of participants	594	254	482	368	320	
		Residential Indoor	17.8 ± 5.1	18.4 ± 3.9	18.0 ± 4.4	18.0 ± 4.2	18.0 ± 4.2	0.42
		Other Indoors	4.8 ± 4.4	4.3 ± 3.2	4.6 ± 3.8	3.6 ± 3.8	3.9 ± 4.0	<0.001
		Transportation	0.8 ± 1.6	0.6 ± 1.3	0.8 ± 1.3	0.8 ± 1.2	0.8 ± 1.2	0.06
		Outdoor	0.6 ± 0.6	0.7 ± 0.7	0.6 ± 0.6	1.6 ± 1.5	1.3 ± 1.7	<0.001
**Gender**	Male	Number of participants	3796	980	1502	1078	1052	
		Residential Indoor	13.5 ± 4.9	14.0 ± 4.7	14.1 ± 5.0	14.7 ± 5.0	15.2 ± 4.6	<0.001
		Other Indoors	8.2 ± 4.4	7.9 ± 4.1	7.9 ± 4.4	6.8 ± 4.6	6.5 ± 4.3	<0.001
		Transportation	1.8 ± 2.1	1.6 ± 1.8	1.5 ± 1.5	1.4 ± 1.3	1.3 ± 1.4	<0.001
		Outdoor	0.5 ± 0.5	0.6 ± 0.6	0.5 ± 0.5	1.1 ± 1.4	1.0 ± 1.7	<0.001
	Female	Number of participants	4276	1022	1702	1174	1120	
		Residential Indoor	15.9 ± 5.0	16.4 ± 4.5	16.2 ± 4.8	16.6 ± 4.9	17.5 ± 4.4	<0.001
		Other Indoors	6.4 ± 4.4	6.0 ± 4.0	6.1 ± 4.2	5.3 ± 4.4	4.7 ± 4.0	<0.001
		Transportation	1.0 ± 1.3	0.9 ± 1.1	1.1 ± 1.2	1.1 ± 1.2	0.9 ± 1.1	0.14
		Outdoor	0.6 ± 0.6	0.7 ± 0.6	0.6 ± 0.6	0.9 ± 1.1	0.9 ± 1.4	<0.001

^a^The data collected in September are compared. The microenvironmental time spent (h) is given as arithmetic mean (AM) ± standard deviation (SD).

^b^Statistical significance of the differences in time spent within each microenvironment from 2004 to 2022, as determined by ANOVA test.

The general trend in time-location profiles within the four microenvironments from 2004 to 2022 were similar across most age groups and in both genders. However, the proportion of adolescents in residential indoors abruptly decreased (>75% to <25%) from 7:30 am to 8:30 am (Fig. S2). In addition, the time of day spent by >75% of adolescents in residential indoors increased from 2004 (10:00 pm to 7:30 am) to 2022 (8:30 pm to 8:00 am). The time of day spent by late-adulthoods in residential indoors did not decrease as much as other age groups from 2004 to 2022. In particular, the proportion of late-adulthoods in residential indoors was higher (>40%) than that of other age groups from 10:30 am to 3:00 pm (Fig. S3). The proportion of adolescents in other indoors abruptly increased (<25% to >75%) from 7:00 am to 9:00 am. However, the time of day spent by >25% of adolescents in other indoors gradually decreased from 2004 to 2022. From 11:00 am to 4:00 pm, the proportion of late-adulthoods in other indoors was lower (<50%) than that in the other age groups (>50%). Unlike other age groups, the time-location profiles of adolescents and late-adulthoods did not exhibit distinct peaks from 7:30 am to 9:30 am and 3:00 pm to 8:00 pm. For adolescents, an abrupt peak (>20%) in time spent outdoors was observed from 7:00 am to 9:00 am.

### TAP trends by work status and day type from 2004 to 2022

The time spent in four microenvironments by different work status and day type from 2004 to 2022 is presented in Table 4. The residential indoor time spent by the employed significantly increased from 12.9 ± 4.6 h in 2004 to 13.5 ± 4.5 h in 2009 (*p* < 0.05) and from 13.4 ± 4.5 h in 2014 to 14.7 ± 4.2 h in 2022 (*p* < 0.001); but those of the unemployed stayed relatively stable from 17.6 ± 4.2 h 2009 to 17.3 ± 4.4 h in 2019 (*p* > 0.1). The transit time spent by the employed decreased constantly from 1.9 ± 2.0 h in 2004 to 1.4 ± 1.2 h in 2022 (*p* < 0.05), whereas that spent by the unemployed were indifferent from 0.8 ± 1.3 h in 2004 to 0.9 ± 1.2 h in 2022 (*p* > 0.05). The residential indoor time spent during weekdays was significantly longer in 2009 (14.3 ± 4.7 h; *p* < 0.05) and 2019 (14.6 ± 4.5 h; *p* < 0.01) than in 2004, 2014, and 2022 (14.0 ± 4.8 h, 14.0 ± 4.6 h, and 14.1 ± 4.0 h, respectively); however, the residential indoor time spent during weekends constantly increased from 16.0 ± 5.3 h in 2004 to 17.4 ± 4.4 h in 2022 (*p* < 0.05), except from 16.7 ± 4.6 h in 2009 to 16.7 ± 4.8 h in 2014 (*p* = 0.125). The other indoor time spent during weekdays was significantly shorter in 2009 (7.6 ± 4.1 h; *p* < 0.05) and 2019 (7.0 ± 4.2 h; *p* < 0.01) than in 2004, 2014, and 2022 (8.0 ± 4.3 h, 8.1 ± 4.1 h, and 8.0 ± 3.8 h, respectively), whereas that during weekends was significantly shorter in 2009 (5.5 ± 3.9 h; *p* < 0.05) and 2022 (3.9 ± 3.7 h; *p* < 0.05) than in 2004, 2014, and 2019 (6.1 ± 4.5 h, 5.7 ± 4.1 h, and 4.4 ± 4.3 h, respectively).

**Table 4:** Descriptive statistics of the microenvironmental time spent by work status and day type from 2004 to 2022.

Group		Microenvironment	Year^a^					*p*-value^b^
			2004	2009	2014	2019	2022	
Work status	Employed	Number of participants	4428	1142	1832	1348	1384	
		Residential Indoor	12.9 ± 4.6	13.5 ± 4.5	13.4 ± 4.5	14.5 ± 4.7	14.7 ± 4.2	<0.001
		Other Indoors	8.7 ± 4.2	8.2 ± 4.0	8.6 ± 4.0	7.1 ± 4.4	7.0 ± 4.0	0.001
		Transportation	1.9 ± 2.0	1.7 ± 1.9	1.6 ± 1.4	1.4 ± 1.3	1.4 ± 1.2	<0.001
		Outdoor	0.5 ± 0.5	0.7 ± 0.6	0.5 ± 0.5	1.0 ± 1.3	0.9 ± 1.8	<0.001
	Unemployed	Number of participants	3644	860	1372	904	788	
		Residential Indoor	17.0 ± 4.8	17.6 ± 4.2	17.4 ± 4.4	17.3 ± 4.4	17.7 ± 4.4	<0.001
		Other Indoors	5.5 ± 4.2	4.9 ± 3.7	5.2 ± 3.8	4.4 ± 3.9	4.2 ± 4.0	<0.001
		Transportation	0.8 ± 1.3	0.7 ± 1.1	0.9 ± 1.2	1.0 ± 1.2	0.9 ± 1.2	0.008
		Outdoor	0.7 ± 0.6	0.8 ± 0.7	0.6 ± 0.6	1.4 ± 1.4	1.3 ± 1.9	<0.001
Day type	Weekdays	Number of participants	4849	1190	1898	1372	1085	
		Residential Indoor	14.0 ± 4.8	14.3 ± 4.7	14.0 ± 4.6	14.6 ± 4.5	14.1 ± 4.0	<0.001
		Other Indoors	8.0 ± 4.3	7.6 ± 4.1	8.1 ± 4.1	7.0 ± 4.2	8.0 ± 3.8	<0.001
		Transportation	1.4 ± 1.7	1.4 ± 1.7	1.3 ± 1.3	1.3 ± 1.3	1.2 ± 1.0	<0.001
		Outdoor	0.6 ± 0.6	0.8 ± 0.7	0.6 ± 0.6	1.1 ± 1.3	0.8 ± 1.1	<0.001
	Weekends	Number of participants	3223	812	1306	880	1087	
		Residential Indoor	16.0 ± 5.3	16.7 ± 4.6	16.7 ± 4.8	17.2 ± 4.9	17.4 ± 4.4	<0.001
		Other Indoors	6.1 ± 4.5	5.5 ± 3.9	5.7 ± 4.1	4.4 ± 4.3	3.9 ± 3.7	<0.001
		Transportation	1.4 ± 1.8	1.1 ± 1.6	1.2 ± 1.4	1.2 ± 1.3	1.3 ± 1.4	<0.001
		Outdoor	0.5 ± 0.6	0.7 ± 0.6	0.5 ± 0.5	1.2 ± 1.5	1.4 ± 2.3	<0.001

^a^The data collected in September are compared. The microenvironmental time spent (h) is given as arithmetic mean (AM) ± standard deviation (SD).

^b^Statistical significance of the differences in time spent within each microenvironment from 2004 to 2022, as determined by ANOVA test.

The general trend in time-location profiles within the four microenvironments from 2004 to 2022 were similar among most groups. However, during weekdays, the proportion of participants in residential indoors from 12:00 am to 6:00 am gradually increased from 2004 to 2022, reaching 99% in 2022 (Fig. S4). In addition, in 2022, the proportion of participants in residential indoors from 10:00 am to 6:00 pm notably decreased. Specifically, during this period, the proportion of participants dropped to <10%, which was less than half of that observed from 2004 to 2019 (20%). In contrast, during weekdays in 2022, the proportion of participants in other indoors from 10:00 am to 6:00 pm showed the greatest increase. The proportion of participants in other indoors reached >70% in 2022, which was higher than that observed from 2004 to 2019 (<70%).

## Discussion

### Temporal evolution in the TAPs of Seoul population from 2004 to 2022

The time spent by the Seoul population in the four microenvironments varied significantly from 2004 to 2022. Specifically, residential indoor and outdoor times increased, whereas other indoor and transit times decreased. Shifts in microenvironmental times can influence the risk of exposure to environmental toxicants [8]. Previous studies have utilized TAP data for exposure modeling. Guak et al. (2021) applied TAP data from 2014 with PM_2.5_ concentrations measured from 2017 to 2018 for the Korea Simulation Exposure Model for PM_2.5_ version 2 [2]. Burke et al. (2001) matched the microenvironmental PM_2.5_ data measured from 1992 to 1993 with the 1990 U.S. census demographic data for the Stochastic Human Exposure and Dose Simulation model [3]. Similarly, the Indoor Air and Exposure Assessment Study and Exposure Assessment of Particulate Matter, and the EXPOLIS model applied TAP data from different periods along with microenvironmental parameters derived from multiple studies [18, 19]. The models often combined the TAP data from one year with the microenvironmental data from another. However, the findings of this study imply that the population’s TAP can change significantly over time. Therefore, TAPs should align with the concurrent microenvironmental data to accurately estimate exposure models.

The time-location profiles of the Seoul population in the four microenvironments changed from 2004 to 2022. In particular, the time of the day spent in other indoors and in transits began later in the morning and ended earlier in the evening; however, the opposite was true for the time of the day spent in residential indoors and outdoors. Shifts in time-location profiles may indicate changes in the duration and intensity of exposure to environmental contaminants. Contaminants and exposure pathways vary across microenvironments, covering inhalation, dermal absorption, and ingestion. Consequently, risk profiles can differ depending on the specific microenvironment and an individual’s activity pattern. Ultraviolet (UV) radiation is known to be the strongest from 10:00 am to 4:00 pm; therefore, spending more time outdoors during these hours could increase UV exposure [20]. In Korea, the ambient PM_10_ concentration was the highest from 8:00 am to 11:00 am, while the levels of ozone (O_3_), nitrogen oxide (NO_x_), and volatile-organic compounds (VOCs) were the highest from 12:00 pm to 7:00 pm [21]. Commencing activities in other indoors or transits at a delayed time in the morning and ending them earlier in the evening may mitigate inhalation exposure to airborne contaminants.

Shifts in the TAPs in this study may account from the changes in human lifestyles and work paradigms. On February 18, 2010, the flexible work system plan was finalized at the national employment strategy meeting in Korea [22]. Employees’ monthly working hours decreased from 180.8 h in 2011 to 154.9 h in 2022, with a notable decline of 10.7 h from 2020 to 2021 [23]. The number of remote workers significantly increased, especially from 95,000 in 2019 to 1.14 million people in 2021, which may indicate a change in the traditional face-to-face interaction culture [24]. The reduced time spent in other indoors and in transits may refer to the decrease in labor hours [25]. Such implies that policy changes could influence how individuals allocate their time, affecting potential exposures to environmental pollutants: if the time-locations of individuals are shifted towards spending more time at residential indoors due to flexible work policies, exposure to in-home pollutants could significantly increase while those in other indoors, transit, and outdoor may decrease. Consequently, comprehensive understanding of the TAP shifts and their relationship to policy changes could aid in developing guidelines for establishing environmental health policies, ensuring that these measures are adapted for reducing environmental pollutant exposures in consideration of the evolving human activity patterns. However, considering the unique characteristics of each individual, it is difficult to conclude in this study, the determinants associated with changes in the TAPs of the Seoul population. Therefore, further investigation is required.

### Differences in TAP trends by demographic groups

For most demographic groups, the time spent in residential indoors increased from 2004 to 2022, whereas that spent in other indoors decreased. However, the trends among adolescents and late-adulthoods were different, which implies different exposure intensities to environmental pollutants among different groups. For adolescents, the time spent in residential indoor significantly increased in 2022, while that in other indoors decreased. The extended time spent in residential indoors by adolescents may increase their exposure to indoor pollutants such as polybrominated diphenyl ether flame retardants (PBDEs). In, Lim et al. (2014), the average concentration of the total PBDEs in indoor dust was higher in schools (1.06 ng/m^3^) than in homes (0.49 ng/m^3^): despite lower levels in homes, due to increased time spent in residential indoor in this study, adolescents’ exposure to in-home PBDEs could significantly increase [26]. On the one hand, for late-adulthoods, the time spent in outdoors was significantly longer in 2022 than in 2004, 2009, and 2014. In Song et al. (2023), the median concentration of PM_2.5_ during fall was significantly higher in outdoors (16.4 µg/m^3^) than in indoors (6.2 µg/m^3^): due to high outdoor levels and increased time spent in outdoors, late-adulthoods’ exposure to outdoor PM_2.5_ could significantly increase [27].

In addition, TAP trends of males differed from females. Specifically, females spent significantly longer residential indoor time than males, and the opposite was true for other indoors, transits, and outdoors, indicating significant heterogeneity in TAPs across demographic groups. This was consistent with previous studies suggesting potential gender-specific exposure disparities [8, 28]. The distinct differences between the demographic groups in this study underpin the need for different approaches to mitigate environmental pollutant exposures for each group. If a specific demographic group consistently experiences exposure to high levels of toxicants, interventions to improve the air quality of the microenvironment at the most frequented time of day should be prioritized.

The disparities in TAP trends by demographic groups may be attributable to their different physiological and social characteristics. Adolescents usually prepared for school from 7:00 am to 9:00 am [29]. They typically engaged in physical activities from 12:00 pm to 3:00 pm at school and from 3:00 pm to 6:00 pm outside school [29]. In contrast, late-adulthoods had a more irregular daily routine due to reduced social commitments. For retirees, the average time spent on moderate-intensity leisure activities was up to 31–42 min/week [30]. Retirees’ sedentary activities increased up to 40.5 min/day, which was more than double (*p* < 0.0001) that of the employed [31]. Gender disparities were primarily accounted by gaps in labor market and travel duration [32–34]. In those studies, despite the increased participation of females in the labor market, a gender gap persisted, and the travel duration was shorter than that of males.

### Differences in TAP trends by work status and day type

The residential indoor time spent by the unemployed was significantly longer than that spent by the employed, and the opposite was true for those in other indoors and transits. Engaged in work and commuting activities, employed participants may have more constraints on social engagement than unemployed participants. Employed participants followed a structured routine, spending less time at home and more time at work or in transit than the unemployed [35]. Although precise information on the participants’ family members was limited in this study, housewives’ schedules may be largely influenced by childcare duties [35]. This may lead to reduced working hours and increased house chore times [36]. In addition, student’s schedule is confined to school and home routines [29].

The time spent in residential indoors during weekdays did not show a linear trend from 2004 to 2022, whereas that spent during weekends increased constantly. In contrast, the transit time spent during weekdays decreased constantly from 2004 to 2022, whereas that during weekdays fluctuated. The differences between the day types in this study coincided with the results of a study conducted in California [37]. In that study, younger adults (<55 years) and children (<8 years) engaged in more sedentary activities in residential indoors during weekends.

During weekdays, the proportion of participants in residential indoors increased the most in 2022, whereas the opposite was true for other indoors. More residential indoor time and less other indoor time in this study could be from the changes occurred during COVID-19. In a Swedish report, 40% of adolescents stated that their exercise programs at home had increased [38]. In a cohort study in Stockholm, the sedentary time spent by the participants significantly increased compared to that in 2019, whereas their physical activity time decreased by 4.2% [39]. However, since the direct effect of COVID-19 on the TAP shifts had not been analyzed in this study, detailed examination of its impact is required in future studies.

### TAP shifts and implications for exposure assessment

This study captured TAPs of Seoul population in 10 min interval: the high-resolution of the KOSTAT and KAPEX research data is novel in the context of exposure literature. Previous studies like the RTP Particulate Matter Panel Study and the National Scale Activity Study encoded in the CHAD, or the EXPOLIS had collected diary data with 15-20 min resolutions [7, 19]. The high-resolution data in this study could provide a refined understanding of individual’s time-location and in-depth assessment of actual population exposures. In addition, this study observed significant variations in the TAPs of Seoul’s population from 2004 to 2022—in particular, the degree of variation differed among different sociodemographic groups. The results in this study highlight the potential impact of TAP shifts on exposure assessment. In a previous study that applied the 2004 TAPs of Seoul people, the average PM_2.5_ exposure of Seoul people was estimated to be 26.0 ± 2.7 µg/m^3^ [40]. In that study, the average microenvironmental PM_2.5_ concentration was the highest in other indoors (34.7 ± 62.3 µg/m^3^), followed by transportation (24.2 ± 22.8 µg/m^3^) and residential indoor (23.7 ± 24.1 µg/m^3^). If the TAP results of 2022 in this study is applied, the contribution of PM_2.5_ exposures in other indoors and transportation would decrease due to reduced time spent in those microenvironments, whereas the opposite would be the case for residential indoor—the 24 h PM_2.5_ exposure would decrease, accordingly. Such could vary more across different sociodemographic groups, as TAP shifts occurred differently. However, direct exposure assessment had not been conducted in this study. Therefore, future researches are required to apply these TAP shifts in practical exposure assessments, thereby providing a more comprehensive insight into environmental exposures.

In exposure modeling, TAP information is crucial; however, the data currently collected do not align with the requirements for an advanced population exposure modeling. Such modeling necessitates compilation of a comprehensive and nuanced information on seasonal variations in TAPs, specific temporal-spatial data, exposure-related human activities, and population variability. Considering the significance of seasonal influence, TAP data had been collected during all four seasons in 2022. However, previous investigations were conducted during three seasons in 2014 and 2019, two seasons in 2009, and only during September in 2004. Given the potential seasonal impact on individuals’ exposure, future studies should carefully examine seasonal variations. Additionally, microenvironments were divisible into only four in this study, and the survey questionnaires primarily focused on the participants’ socioeconomic activities. In particular, key daily activities, such as chemical storage or home appliance usage, and detailed information on the specific locations that individuals frequented were overlooked. Therefore, specific microenvironments and exposure-related activities need to be investigated. Lastly, the surveys in this study were conducted on a quintennial basis except 2022, with varying samples sizes. However, the TAPs varied significantly during each investigation, indicating the need for further consideration on the survey period. Although population weights for factors including selection biases, non-responses, and population alignments were considered in each survey, large variations in sample size can still introduce variability; the disparities could potentially affect the stability and comparability of the results, leading to skewed interpretations. Limitations in TAP data could lead to a mismatch with the environmental pollutant data measured during different periods. Given that TAP changes could significantly impact the outcomes of exposure models, a thorough examinations of survey frequency and the appropriate sample size are required in future studies.

## Conclusions

This study observed a significant evolution of TAPs in the Seoul population from 2004 to 2022, with disparities in TAP trends among different sociodemographic groups. These findings highlight the dynamic nature of TAPs and their potential impact on the exposure to environmental pollutants. Substantial variations in TAPs across different microenvironments and sociodemographic groups have significant implications for exposure modeling and risk assessment. The results of this study emphasize the importance of aligning TAP data with concurrent microenvironmental pollutant data. As TAPs continue to evolve, further research is essential to understand and address their implications for public health and environmental policy. In addition, thorough examinations regarding survey seasons, survey items, and investigation frequency are needed for improved exposure assessments in the future.

## Supplementary information

Supplementary information

## Data Availability

All original data used in this study are available upon request.
