# Supplementary material for: Temporal trend of microenvironmental time-activity patterns of the Seoul population from 2004 to 2022 and its potential impact on exposure assessment
Source: J Expo Sci Environ Epidemiol. 2024 Mar 28;35(2):315–24. doi: 10.1038/s41370-024-00662-1 (PMC12009733; doi:10.1038/s41370-024-00662-1)
Supplement: Supplementary file 1 — Supplementary information [file 41370_2024_662_MOESM1_ESM.docx]

**Supplementary materials**

**Table S1.** Sociodemographic related questionnaire categories.

| **Main Category** | **Subcategory** | **Item** | **Main Category** | **Subcategory** | **Item** | **Main Category** | **Subcategory** | **Item** |
| --- | --- | --- | --- | --- | --- | --- | --- | --- |
| **Personal Care Management** | Sleep | Sleeping | **Household Chore Activity** | Food Preparation And Cleanup | Meal Preparation | **Social Activity** | Using Phone For Socializing (Including Text Messages) | Socializing With Family/Relatives |
|  |  | Napping |  |  | Dishwashing, Post-Meal Cleanup |  |  | Socializing With Others |
|  | Eating | Eating With Family |  |  | Making Snacks And Non-Routine Foods (Preserved Foods, Etc.) |  |  | Using Internet For Socializing (Chat, Email, Etc.) |
|  |  | Eating Alone |  | Clothing Management | Laundry And Hanging Clothes |  |  | Visiting Graves, Weeding |
|  |  | Eating With Non-Family |  |  | Clothes Arrangement (Collecting Laundry, Organizing Wardrobe, Etc.) |  |  | Other Socializing Related Activities |
|  |  | Snacks And Beverages |  |  | Ironing, Sewing, Clothes Maintenance |  | General Public Learning | Foreign Language Learning |
|  | Personal Care | Personal Hygiene |  |  | Clothing Repair, Laundry Service |  |  | Computer-Related Learning |
|  |  | Appearance Care (Makeup, Changing Clothes, Etc.) |  |  | Sewing, Knitting |  |  | Certification, Employment-Related Learning (Skills, Techniques, Etc.) |
|  |  | Receiving Beauty Services |  | Cleaning And Organizing | Room/Item Organization |  |  | Hobby-Related Learning |
|  | Health Care (Medical) | Self-Treatment |  |  | Home Cleaning (Sweeping, Wiping) |  |  | Other General Public Learning |
|  |  | Receiving Medical Services |  |  | Other Cleaning And Organizing |  | Media Use | Newspapers |
|  |  | Resting Due To Illness |  | Home Maintenance | Household Tools/Home Diy |  |  | Magazines |
|  | Other | Other Personal Maintenance |  |  | Car Washing, Direct Car Maintenance |  |  | TV |
| **Occupation** | Employed Work And Self-Employment | Main Job |  |  | Other Direct Home Maintenance |  |  | Videos, Dvds, Pmp, Dmb |
|  |  | Side Job |  |  | Household Tools/Home Maintenance Service |  |  | Radio |
|  |  | Break During Work |  |  | Car Washing, Car Maintenance Service |  |  | Cds, Tapes, Mp3, Etc. |
|  |  | Training Related To Work |  |  | Other Home-Related Services |  |  | Internet Information Search (Including Wireless) |
|  |  | Bringing Work Home |  | Purchasing Household-Related Items | Direct Shopping For Household Goods |  |  | Other Internet (Personal Homepage, Blog Management, Movies, Etc.) |
|  |  | Other Work-Related Behavior |  |  | Direct Shopping For Durable Goods (Excluding Household Goods) |  | Religious Activities | Personal Religious Activities |
|  | Unpaid Family Work (Excluding Agriculture, Forestry, Fishing) | Unpaid Family Work (Excluding Agriculture, Forestry, Fishing) |  |  | Non-Store Shopping For Household Goods (Internet, Tv Home Shopping, Etc.) |  |  | Religious Gatherings/Meetings Participation |
|  |  | Unpaid Family Work In Agriculture, Forestry, Fishing |  |  | Non-Store Shopping For Durable Goods (Excluding Household Goods) (Internet, Tv Home Shopping, Etc.) |  |  | Other Religious Related Activities |
|  |  | Unpaid Family Work In Agriculture, Forestry, Fishing |  | Household Management | Household Account Management | **Leisure Activity** | Attending And Participating In Cultural Events | Movies (Theater/Video Room) |
|  |  | Self-Consumption Agriculture, Forestry, Fishing Work |  |  | Home Planning |  |  | Theater, Concerts |
|  |  | Self-Consumption Agriculture, Forestry, Fishing Work |  |  | Bank And Government Office Visits |  |  | Exhibitions, Museums |
|  | Job Seeking | Job Seeking |  | Other Domestic Tasks | Other Domestic Tasks |  |  | Watching Sports Matches |
|  | Purchasing Work-Related Items | Direct Shopping For Work | **Family Caregiving** | Taking Care Of Pre-School Children | Physical Care (Washing, Feeding, Putting To Sleep, Etc.) |  |  | Other Attendance And Cultural Event Participation |
|  |  | Non-Store Shopping For Work (Internet, Tv Home Shopping, Etc.) |  |  | Helping With Study, Reading Books |  | Sports And Outdoor Leisure Activities | Walking, Strolling |
|  | Other Work-Related Behavior | Other Work-Related Behavior |  |  | Playing |  |  | Mountain Climbing, Hiking (Including Forest Bathing) |
| **Education Level** | Learning In Regular Classes | Breaks Between Regular Classes |  |  | Nursing Pre-School Children |  |  | Individual Exercises For Fitness (Calisthenics, Swimming, Gym, Aerobics, Yoga, Etc.) |
|  |  | Self-Study At School |  |  | Other Pre-School Child Care |  |  | Other Team Sports (Basketball, Soccer, Bowling, Billiards, Golf, Badminton, Inline Skating, Etc.) |
|  |  | School Events |  | Taking Care Of School-Aged Children | Helping With Washing, School Commute |  |  | Driving, Picnicking, Sightseeing And Exploring |
|  |  | Other Learning-Related Behavior At School |  |  | Homework And Study Assistance |  |  | Other Outdoor Leisure Activities (Fishing, Camping, Amusement Parks, Etc.) |
|  | Learning Outside Regular Classes |  |  |  | Consultation And School Visits |  | Reading | Computer Games (Including Portable Gaming Devices) |
|  |  | Enrolling In Classes Outside Of Regular Classes |  |  | Nursing School-Aged Children |  |  | Games (Go, Chess, Cards, Children'S Games, Etc.) |
|  |  | Self-Study Outside Of School |  |  | Other School-Aged Child Care |  |  | Other Hobbies (Calligraphy, Crafting, Collecting, Playing Instruments, Etc.) |
|  |  | Other Learning-Related Behavior Outside Of School |  | Spouse Care | Nursing Spouse |  |  | Entertainment (Drinking, Dancing, Karaoke) |
|  | Purchasing Learning-Related Items | Direct Shopping For Learning |  |  | Other Spouse Care |  |  | Smoking |
|  |  | Non-Store Shopping For Learning (Internet, Tv Home Shopping, Etc.) |  | Taking Care Of Parents And Grandparents | Nursing Parents And Grandparents |  |  | Doing Nothing And Resting |
| **Volunteer Activity Or Internship** | Helping Neighbors And Acquaintances | Helping With Income-Generating Activities |  |  | Other Parental And Grandparental Care |  | Purchasing Goods Related To Socializing And Leisure Activities | Direct Shopping For Socializing And Leisure Activities |
|  |  | Helping With Household Activities |  | Taking Care Of Other Family Members | Nursing Other Family Members |  |  | Non-Store Shopping For Socializing And Leisure Activities (Internet, Tv Home Shopping, Etc.) |
|  |  | Other Helping Activities |  |  | Other Care For Other Family Members |  | Other Leisure Related Activities | Other Leisure Related Activities |
|  | Participation Activities | Mandatory Participation Activities |  | Other Family Care Related Behavior | Other Family Care Related Behavior | **Transportation** | Personal Care Related Travel |  |
|  |  | Voluntary Participation Activities |  | | |  | Commuting |  |
|  | Volunteering | Related To National And Local Events |  |  |  |  | Other Work Related Travel |  |
|  |  |  |  |  |  |  | School And Study Related Travel |  |
|  |  | Related To Child Education |  |  |  |  | Household Chore Related Travel |  |
|  |  |  |  |  |  |  | Family Care Related Travel |  |
|  |  | Related To Children, Elderly, Disabled, Etc. |  |  |  |  | Participation And Volunteer Activities Related Travel |  |
|  |  | Helping And Facility Recovery After Disasters |  |  |  |  | Social Activities Related Travel |  |
|  |  |  |  |  |  |  | General Public Learning Related Travel |  |
|  |  | Other Volunteering (Blood Donation, Free Counseling, Etc.) |  |  |  |  | Other Leisure Activities Related Travel (Media, Religion, Watching, Sports, Hobbies, Etc.) |  |
|  |  |  |  |  |  |  | Other Travel |  |
|  |  |  |  |  |  |  | Waiting For Travel |  |

**Table S2.** Summary of the sociodemographic characteristics of the study population investigated in this study.

| **Sociodemographic characteristics** | | **Year** | | | | |
| --- | --- | --- | --- | --- | --- | --- |
|  |  | **2004 (%)**  **(n=8,072)** | **2009 (%) (n=2,002)** | **2014 (%) (n=3,204)** | **2019 (%) (n=2,252)** | **2022 (%) (n=2,172)** |
| **Age** | <19 | 15 | 9 | 12 | 8 | 14 |
|  | 19-29 | 20 | 12 | 14 | 16 | 16 |
|  | 30-49 | 41 | 43 | 36 | 34 | 30 |
|  | 50-64 | 16 | 23 | 23 | 26 | 25 |
|  | >65 | 8 | 13 | 15 | 16 | 15 |
| **Gender** | Male | 47 | 49 | 47 | 48 | 48 |
|  | Female | 53 | 51 | 53 | 52 | 52 |
| **Educational level** | Up to middle school | 28 | 27 | 24 | 17 | 15 |
|  | Up to high school | 36 | 31 | 29 | 25 | 36 |
|  | Up to college or higher | 36 | 42 | 47 | 58 | 49 |
| **Marriage** | No | 37 | 34 | 24 | 33 | 21 |
|  | Yes | 55 | 65 | 73 | 57 | 75 |
|  | Separated | 8 | 1 | 3 | 10 | 4 |
| **Work status** | Employed | 55 | 57 | 57 | 60 | 64 |
|  | Unemployed | 45 | 43 | 43 | 40 | 36 |

**
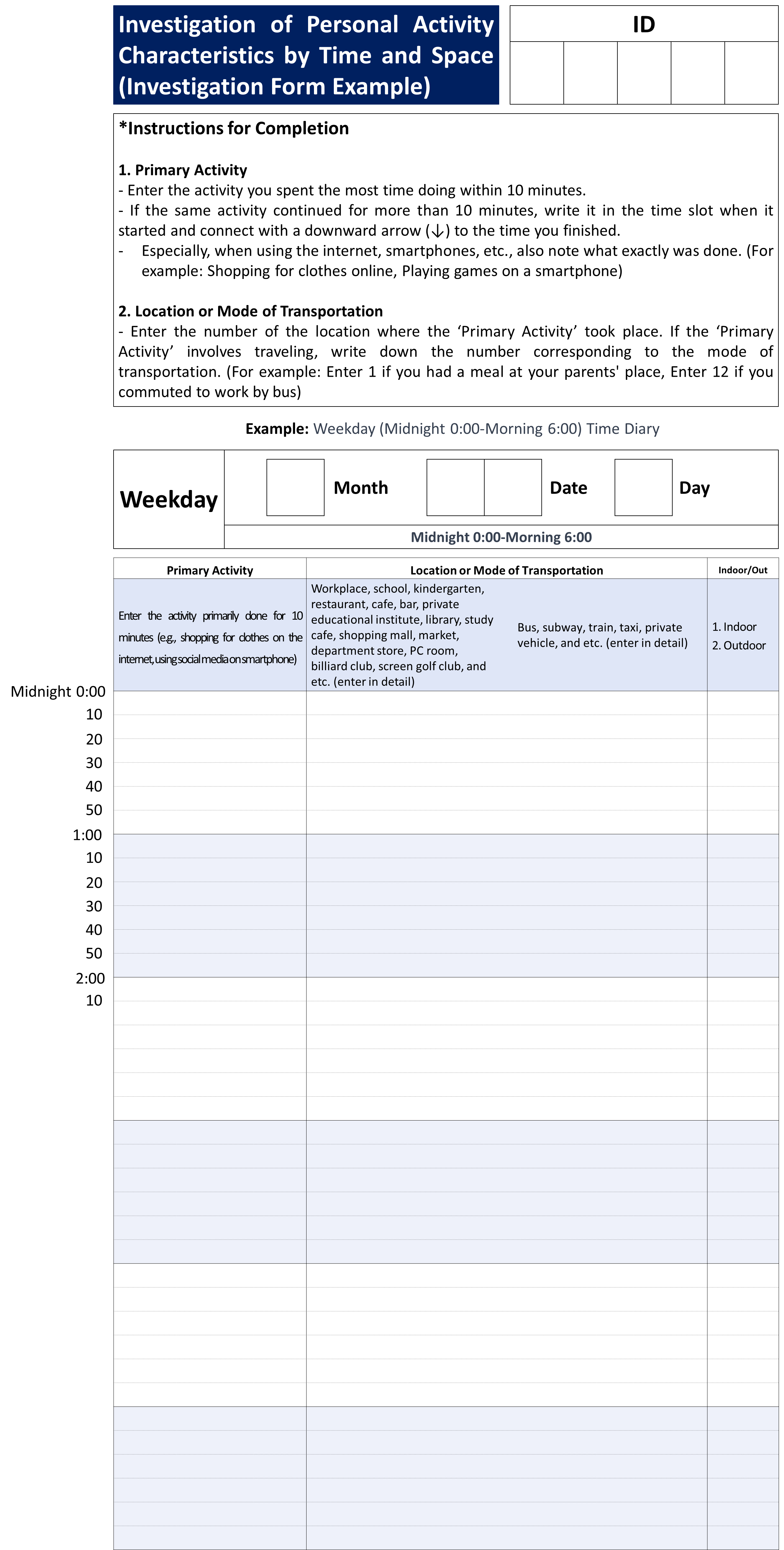
**

**Fig. S1: Schematic diagram of a time-diary.**

**
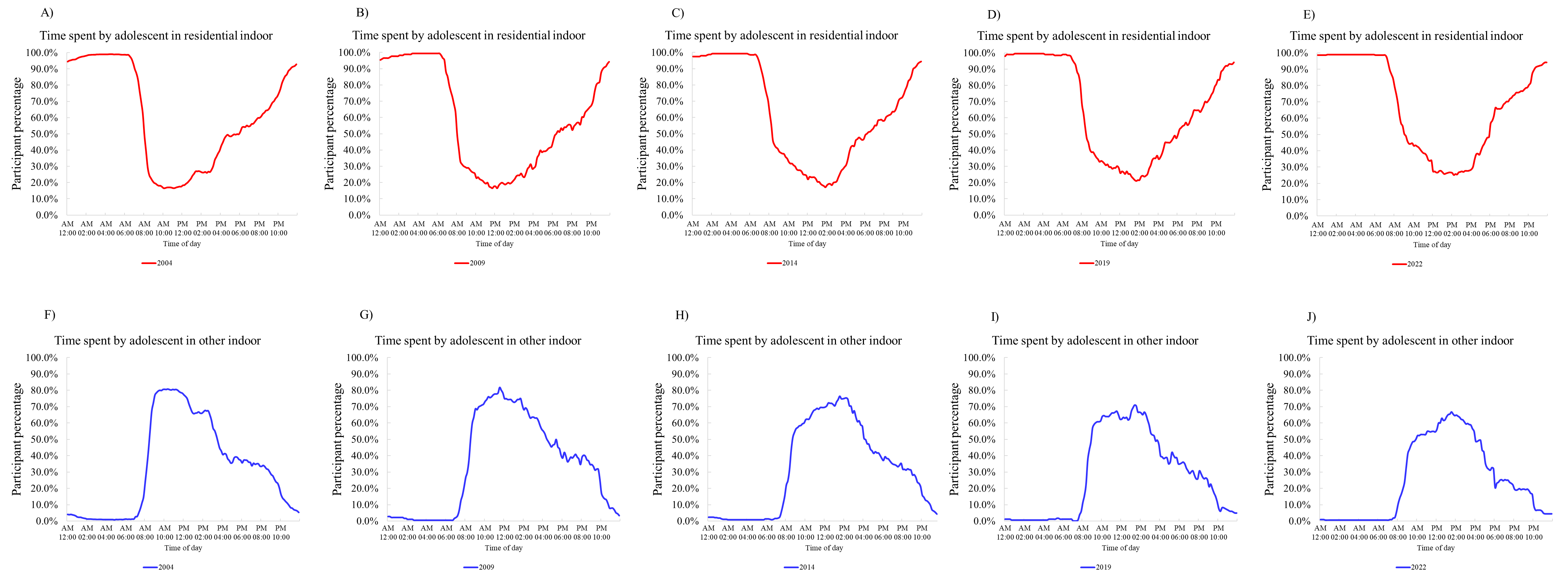
**

**Fig. S2:** **The time-location profiles of adolescents.** A), B), C), D), and E) are the proportions of adolescents (%) in residential indoors during 2004, 2009, 2014, 2019, and 2022, respectively. F), G), H), I), and J) are the proportions of adolescents (%) in other indoors during 2004, 2009, 2014, 2019, and 2022, respectively.

**
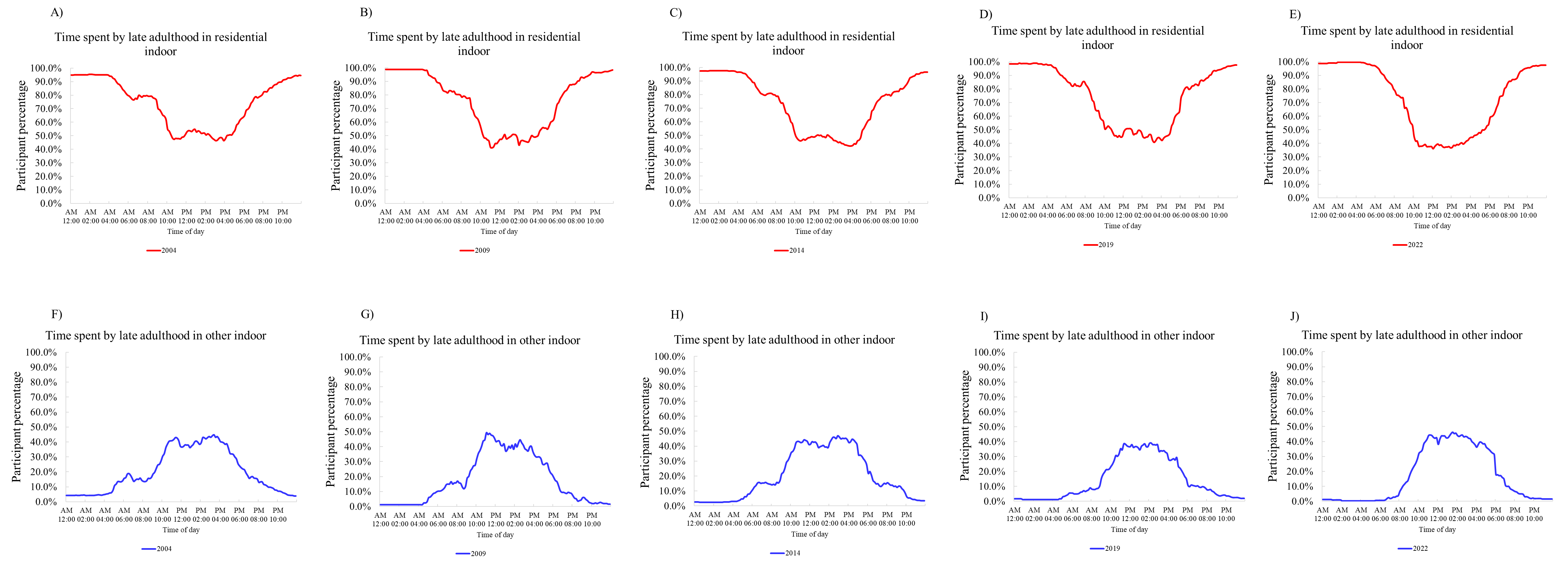
Fig. S3: The time-location profiles of late-adulthoods.** A), B), C), D), and E) are the proportions of late-adulthoods (%) in residential indoors during 2004, 2009, 2014, 2019, and 2022, respectively. F), G), H), I), and J) are the proportions of late-adulthoods (%) in other indoors during 2004, 2009, 2014, 2019, and 2022, respectively.

**
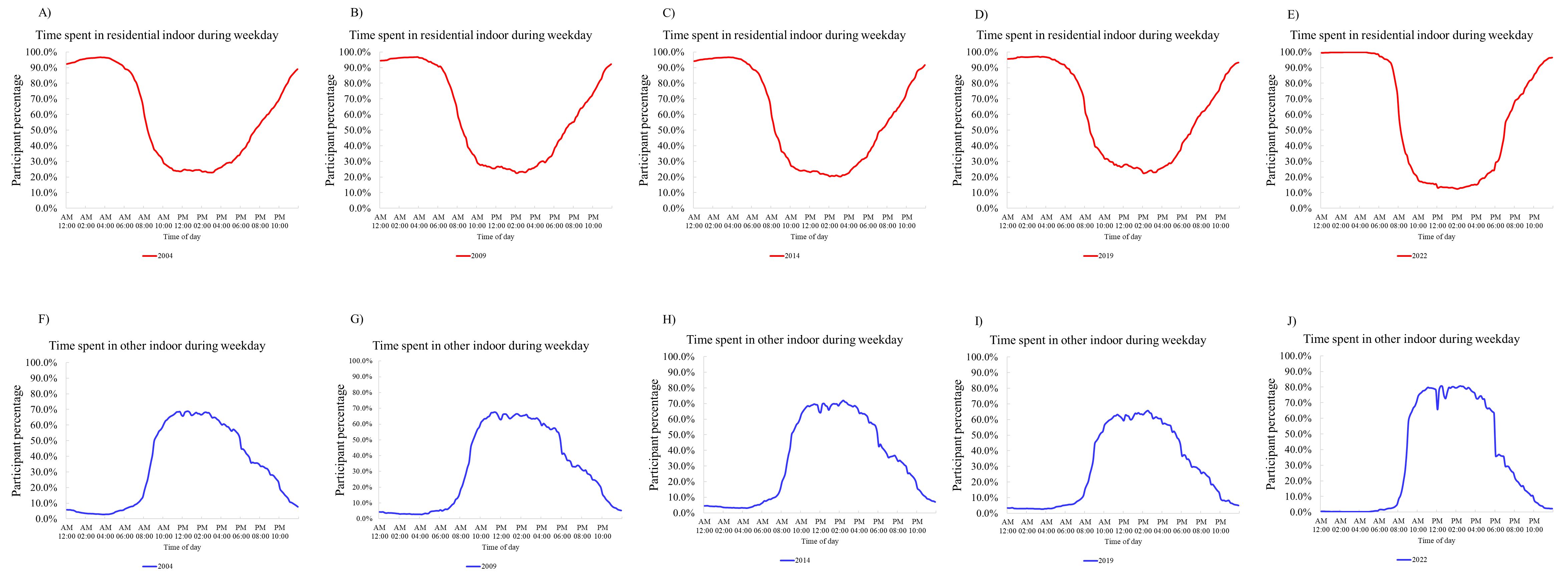
Fig. S4: The time-location profiles of participants on weekdays.** A), B), C), D), and E) are the proportions of participants (%) in residential indoors during 2004, 2009, 2014, 2019, and 2022, respectively. F), G), H), I), and J) are the proportions of participants (%) in other indoors during 2004, 2009, 2014, 2019, and 2022, respectively.
